# Resilience and mental health: A longitudinal cohort study of Chinese adolescents before and during COVID-19

**DOI:** 10.3389/fpsyt.2022.948036

**Published:** 2022-08-17

**Authors:** Wei Shi, Li Zhao, Min Liu, Binxue Hong, Lihua Jiang, Peng Jia

**Affiliations:** ^1^Institute for Disaster Management and Reconstruction (IDMR), Sichuan University, Chengdu, China; ^2^Department of Health Policy and Management, West China School of Public Health and West China Fourth Hospital, Sichuan University, Chengdu, China; ^3^Department of Health Behavior and Social Medicine, West China School of Public Health and West China Fourth Hospital, Sichuan University, Chengdu, China; ^4^School of Resource and Environmental Sciences, Wuhan University, Wuhan, China; ^5^International Institute of Spatial Lifecourse Health (ISLE), Wuhan University, Wuhan, China

**Keywords:** resilience, depression, anxiety, COVID-19, adolescents

## Abstract

**Background:**

The COVID-19 outbreak has resulted in mental health issues, mainly depression and anxiety, prompted by stressors such as the need to maintain social distance, adapting to quarantine, and lockdown policies. Resilience may be vital in protecting individuals from mental disorders. However, few studies have examined the longitudinal relationships between resilience and mental disorders (i.e., depression and anxiety) among adolescents before and during the COVID-19 pandemic.

**Objective:**

This study aimed to examine the association between resilience, depression, and anxiety among Chinese adolescents before and during COVID-19 using a longitudinal cross-lagged model.

**Methods:**

A total of 7,958 Chinese adolescents completed a baseline survey in the month before COVID-19 and were followed up after the COVID-19 lockdown. Structural equation modeling analyses were applied to evaluate the associations between resilience, depression, and anxiety after controlling for three covariates (i.e., gender, age, and COVID-19 effect).

**Results:**

A higher level of resilience before COVID-19 significantly predicted decreased severity of depression and anxiety after the lockdown. Moreover, the mean level of resilience and prevalence of mental disorders (i.e., depression and anxiety) among Chinese adolescents decreased after the lockdown. These findings suggest resilience is a vital protective factor against depression and anxiety among adolescents. Furthermore, younger participants and those less affected by the pandemic could be more resilient. No significant link was found between gender and resilience in the second wave.

**Conclusions:**

Resilience is an essential protective factor for reducing mental disorders among Chinese adolescents exposed to COVID-19. Resilience-related interventions should be developed to efficiently promote mental health recovery among youth during pandemics.

## Introduction

*Resilience* may be vital in protecting individuals from common mental disorders such as depression and anxiety during COVID-19. Resilience is a series of internally psychological and adaptive characteristics that protect individuals in adverse, stressful, and difficult circumstances (1, 2). Previous studies have indicated that resilience and related psychological traits, such as constructive stress management strategies, optimism, and hopeful self-belief, were linked to a reduced risk of mental disorders (e.g., depression and anxiety) during public health emergencies (3). For example, a cross-sectional study with 296 COVID-19 patients demonstrated that resilience was significantly negatively linked with depression and anxiety (4). Similarly, a study with 255 nurses revealed a negative association between mental disorders (i.e., depression and anxiety) and resilience (5). Moreover, another study with 518 adults in Turkey during COVID-19 reported that a higher level of resilience was associated with lower severity of depression (6). However, most prior studies were cross-sectional, lacking consistent evidence clarifying the protective function of resilience against mental disorders [e.g., (4, 7)]. Longitudinal studies may thus provide stronger evidence of this association (3).

Adolescence is a sensitive and vital stage of physical and psychological development. An emerging consensus indicates that the COVID-19 pandemic has resulted in mental health problems provoked by psychological challenges, such as an enduring economic slump, livelihood pressure, maintaining social distance, and adapting to quarantine and closure policies (8, 9). During COVID-19, adolescents may have been vulnerable to common mental disorders, such as depression and anxiety (8). According to the World Health Organization (10), one in seven (approximately 14%) adolescents aged 10–19 are estimated to be experiencing major mental health issues globally. The outbreak and persistence of COVID-19 could psychologically burden adolescents (9). A study with 8,140 Chinese adolescents indicated an increased prevalence of depression and anxiety after the start of the pandemic (9). However, some studies reported that the prevalence of mental disorders (e.g., depression, anxiety) decreased in Switzerland (11), England (12), and the United States (13) in adolescents exposed to COVID-19. Longitudinal research could examine these contradictory findings and further clarify trends in mental health among adolescents before and during COVID-19.

Certain theories propose that resilience facilitates youth mental health. According to the resilience theory, two major perspectives (i.e., trait-oriented and process-oriented perspectives) explain why some adolescents develop into psychologically adaptable and healthy adults in the face of hardship through the cultivation of resilience (14). The trait-oriented perspective regards resilience as a personal characteristic that helps youths to overcome difficulties *via* optimal self-adjustment (15). The process-oriented perspective defines resilience as resulting from the interaction between individuals and the environment, suggesting that individuals develop it by successfully overcoming the adverse impact of exposure to danger and traumatic events (16). Additionally, the protective model, rooted in resilience theory, explains the preventive role of related emotional regulation skills. These skills act as buffers against the adverse effects of stressors and internalizing mental problems (1, 17). Moreover, based on resilience theory, some studies have confirmed the protective function of resilience in reducing mental health issues (e.g., depression and emotional problems) in adversity [e.g., (7, 18–21)]. Consequently, resilience may be vital in protecting individuals against the negative influence of traumatic events and mental problems. Yet studies exploring the longitudinal effects of resilience on mental health among adolescents exposed to COVID-19 are lacking.

### The current study

Most studies described above only applied a cross-sectional design to examine the concurrent links between variables [e.g., (4)]. Some previous studies among adolescents exposed to disasters suggested cross-sectional associations between resilience and internalizing mental health problems, such as depression, anxiety, and emotional distress (5, 21, 22). However, these studies did not simultaneously explore the links, and the longitudinal associations between these variables remain unclear. Additionally, many studies focused on protective factors against mental disorders during COVID-19 among adults only (5, 23). Therefore, our study used a time-lagged study design to explore the prospective bidirectional links between resilience, depression, and anxiety during the COVID-19 pandemic, focusing on Chinese adolescents to address the gaps in the literature described above.

We conducted a two-wave longitudinal survey at a 6-month interval to explore the relationships between resilience, depression, and anxiety *via* a structural equation model (SEM). Furthermore, we tracked the trends in resilience, depression, and anxiety among the survey participants. Referring to previous research (4, 5), we hypothesized that resilience at Time 1 (T1) would be significantly negatively associated with depression and anxiety 6 months later (at Time 2; T2). We explored the remaining bidirectional links, mental health trends, and resilience flexibly without addressing any set hypotheses.

## Methods

### Dataset

This study's dataset was obtained from the Chengdu Positive Child Development (CPCD) survey. This survey aimed to investigate mental health and protective factors longitudinally among Chinese youth before and after COVID-19 (24). The CPCD survey randomly recruited participants from five schools in Chengdu, including three senior high schools and two secondary schools. A self-report questionnaire was used to collect data during the COVID-19 lockdown in China. Time 1 (T1) data collection occurred from December 23, 2019 to January 13, 2020 (before the pandemic began). Time 2 (T2) data collection was performed from June 16 to July 8, 2020 (during the pandemic). Thus, 6 months elapsed between the survey waves. A total of 10,370 students were invited to complete the survey, and 8,749 provided valid responses at T1 (effective response rate: 84.37, 48.38 female). Follow-up included 7,958 participants at T2 (effective response rate: 76.74%). A total of 791 participants were lost to follow-up at T2. The research protocol was reviewed and approved by the Medical Ethics Committee of Sichuan University (Register no. K2020025) and the school administrations where data were collected.

### Measures

#### Anxiety

A nine-item subscale of the Screen for Child Anxiety Related Emotional Disorders (SCARED) was used to evaluate generalized anxiety disorder for the last 3 months in both survey waves (25). Each item used a three-point Likert scale (0 = never, 2 = often). A sample item is “I worry about other people liking me.” Summing the scores of all items represents the anxiety symptoms levels. A higher score denotes more severe anxiety. A total score over nine indicates the presence of an anxiety disorder (25). The Chinese version of SCARED used in this study has good validity and reliability according to prior research [e.g., (26)]. The scale's reliability was good in both survey waves (T1 Cronbach's α = 0.86; T2 Cronbach's α = 0.88).

#### Depression

The 20-item Center for Epidemiologic Studies Depression Scale (CES-D) was used to examine depressive symptoms over the past week in both study waves (27). Each item is rated on a four-point Likert scale (0 = not at all, 3 = a lot). Four reverse-scored items are included (i.e., items 4, 8, 12, and 16). A sample item is “I was bothered by things that usually don't bother me.” The total item score represents the severity of depressive symptoms. Higher scores indicate more severe depression. A total score over 15 is the cut-off for a depression diagnosis (27). The Chinese CES-D has shown excellent reliability and validity in prior research [e.g., (28)]. This scale displayed good reliability in both survey waves (T1 Cronbach's α = 0.87; T2 Cronbach's α = 0.89).

#### Resilience

A six-item subscale of the Chinese Positive Youth Development Scale (CPYDS) was used to examine resilience in both study waves (29). Each item was rated on a six-point Likert scale (1 = extremely disagree, 7 = extremely agree). A sample item is “When I face difficulty, I will not give up easily.” The total item scores indicated the resilience level. A higher score represented stronger resilience. Some studies have demonstrated that CPYDS has good reliability and validity [e.g., (30)]. The subscale's reliability was good in both survey waves (T1 Cronbach's α = 0.82; T2 Cronbach's α = 0.87).

#### Participant characteristics

Demographic information collected included age, gender, and COVID-19 effect. COVID-19 effect refers to the individual's perception of how strongly COVID-19 influenced their daily and social lives. Nine items were created to examine the COVID-19 effect in the T2 survey based on context and prior research (31). Four items assessed perceived severity, menace, infection risk, and prophylaxis against COVID-19, rated on a four-point Likert scale (1= not at all, 4 = extremely severe). In addition, one dichotomous item confirmed an identified case of infection in a family (1 = no; 2 = yes). Four questions examined the perceived influence of COVID-19 on study, diet, socializing, and recreational activities. These items were rated using a four-point Likert scale (1 = none, 4 = extremely influenced). The total score of all items indicated the degree of COVID-19 effect. A higher score represented a stronger influence.

### Statistical analysis

The data analysis of the longitudinal study consisted of four steps using statistical software programs SPSS Version 24.0 (32) and AMOS 23.0 (33). First, descriptive analyses and a correlation matrix of the study variables were performed in SPSS. Second, an independent-samples *t*-test was performed to examine differences between the 7,958 participants and 791 respondents lost to follow-up at the T2 study. A paired-samples *t*-test examined the differences in depressive and anxious symptoms between waves. Third, a multiple collinearity test evaluated collinearity problems among all study variables. An invariance measurement evaluated the stability of the cross-lagged model. Finally, structural equation modeling was used to assess the goodness-of-fit of the longitudinal cross-lagged model and significant relations between the main variables (resilience, depression, and anxiety at T1 and T2) after controlling for three covariates, namely age, sex, and COVID-19 effect), using 5,000 bootstrapped replications.

Regarding inter-correlations between variables (34), an absolute coefficient value between 0.50 and 1 is considered to indicate a high correlation level. A coefficient's absolute value between 0.30 and 0.49 indicates a moderate correlation. An absolute coefficient value below 0.29 represents a small correlation. Moreover, previous research recommends using specific goodness-of-fit indexes to evaluate the model quality and significant paths (35). These indexes include χ^2^/*df* (degree of freedom), root mean square error of approximation (RMSEA), standardized root mean square residual (SRMR), normed fit index (NFI), Tucker & Lewis index (TLI), and comparative-fit index (CFI). If χ^2^/*df* is below 5.0, NFI, TLI, and CFI are above 0.95, and SRMR and RMSEA are below 0.05, the longitudinal cross-lagged model indicates a good fit.

## Results

### Participant characteristics

Participants' demographic information is displayed in Table 1. Our study included 7,958 participants who completed the two-wave survey, 48.33% were females, and the mean age was 11.74 years (range: 7–17, SD = 2.15). No significant differences were detected between valid participants and those lost to follow-up in any variables (*p* > 0.001) except anxiety and age. More severe anxiety symptoms and older age were associated with a higher probability of dropping out of the follow-up study.

**Table 1:** Demographics (*N* = 7,958).

**Variable**	**(*M, SD, n*, %)**
**Age (7**–**17)**	*M* = 11.74, SD = 2.15
**Gender**	
Male	4,112 (51.67%)
Female	3,846 (48.33%)
**COVID-19 effect**	*M* = 21, *SD* = 4.05
**Resilience** ^*^	
T1	*M* = 31.91, *SD* = 4.65
T2	*M* = 31.55, *SD* = 5.24
**Depression** ^**ns**^	
T1	*M* = 14.40, *SD* = 10.16
	*n*_Yes_ = 3,078 (38.68%)
	*n*_no_ = 4,880 (61.32%)
T2	*M* = 14.36, *SD* = 10.62
	*n*_Yes_ = 2,924 (36.74%)
	*n*_no_ = 5,034 (63.26%)
**Anxiety** ^*^	
T1	*M* = 3.71, *SD* = 3.96
	*n*_Yes_ = 1,036 (13.02%)
	*n*_no_ = 6,922 (86.98%)
T2	*M* = 3.33, *SD* = 4.07
	*n*_Yes_ = 1,016 (12.77%)
	*n*_no_ = 6.942 (87.23%)

(1) M, mean; SD, standard deviation; n_yes_, number of participants with anxiety or depressive symptoms; n_no_, number of participants without anxiety or depressive symptoms; T1, Time 1; T2, Time 2; (2) Dichotomous cut-off scores: depressive symptoms ≥ 15, anxiety symptoms ≥ 9; (3) ^*^p < 0.001 (paired-sample t-test across waves); ns, not significant (p = 0.74).

### Inter-correlations and invariance measurement

Table 2 displays the means, standard deviations, and intercorrelations of all study variables. When controlling for gender, age, and COVID-19 effects, most study variables showed a relatively moderate or high correlation with each other, apart from T1 resilience with T1 anxiety (*r* = −0.27, *p* < 0.001), with T2 anxiety (*r* = −0.21, *p* < 0.001), and with T2 depression (*r* = −0.27, *p* < 0.001), respectively; these variables exhibited comparatively low correlations. Similarly, T2 resilience had a lower correlation with T1 anxiety (*r* = −0.27, *p* < 0.001). Additionally, resilience at T1 and T2 showed significantly negative longitudinal correlations with both anxiety and depression.

**Table 2:** Means, standard deviations, and correlations between study variables (*N* = 7,958).

**#**	**Variables**	**M ±SD**	**1**	**2**	**3**	**4**	**5**	**6**
1	T1 Anxiety	3.71 ± 3.96	/	0.49^*^	0.61^*^	0.41^*^	−0.27^*^	−0.27^*^
2	T2 Anxiety	3.33 ± 4.07	0.52^*^	/	0.40^*^	0.62^*^	−0.21^*^	−0.32^*^
3	T1 Depression	14.40 ± 10.16	0.61^*^	0.41^*^	/	0.52^*^	−0.36^*^	−0.34^*^
4	T2 Depression	14.36 ± 10.62	0.43^*^	0.64^*^	0.53^*^	/	−0.27^*^	−0.41^*^
5	T1 Resilience	31.91 ± 4.65	−0.28^*^	−0.23^*^	−0.36^*^	−0.29^*^	/	0.39^*^
6	T2 Resilience	31.55 ± 5.24	−0.29^*^	−0.34^*^	−0.34^*^	−0.42^*^	0.40^*^	/

(1) The number in lower left is the correlation coefficient without controlling for age and gender; the number in the top right is the correlation coefficient after controlling for age, gender, and COVID-19 exposure. (2) ^*^ p < 0.001. (3) Correlation interval: High degree: coefficients value between ±0.50 and ± 1; Moderate degree: coefficients value between ±0.30 and ±0.49; Low degree: coefficients value below +0.29.

Based on recommendations of prior researchers [e.g., (36)], stability coefficients were used to assess the invariance measurement of the study model. Stability coefficients above 0.4 indicate measurement invariance in the longitudinally cross-lagged model. The stability coefficients of resilience, anxiety, and depression across the two waves were 0.47, 0.51, and 0.59, respectively. This result confirmed the measurement invariance of the cross-wave study model.

### Collinearity test

Previous research states that a multicollinearity problem exists when the variance inflation factor (VIF) is above 4.0 and tolerance is below 0.25 (37). The results of the multiple collinearity test indicated that the VIF and tolerance of resilience (VIF = 1.16, tolerance =0.87), depression (VIF = 1.69; tolerance =0.59), and anxiety (VIF = 1.60; tolerance =0.63) at T1 were within limits. Consequently, there is a low likelihood of multicollinearity problems among study variables.

### Prevalence of depression and anxiety

Table 1 displays the mental health status of study participants in both waves. The prevalence of depression was 38.68% (*n* = 3,078) at T1 and 36.76% (*n* = 2,924) at T2. The prevalence of anxiety was 13.02% (*n* = 1,036) at T1 and 12.77% at T2 (*n* = 1,016). There was a declining trend in the prevalence of depression and anxiety among sampling participants from before to during the pandemic. Moreover, paired-samples *t*-test results showed that anxiety prevalence and mean level of resilience significantly decreased among participants across waves (*p* < 0.001).

### Cross-lagged path model

**Figure 1:**
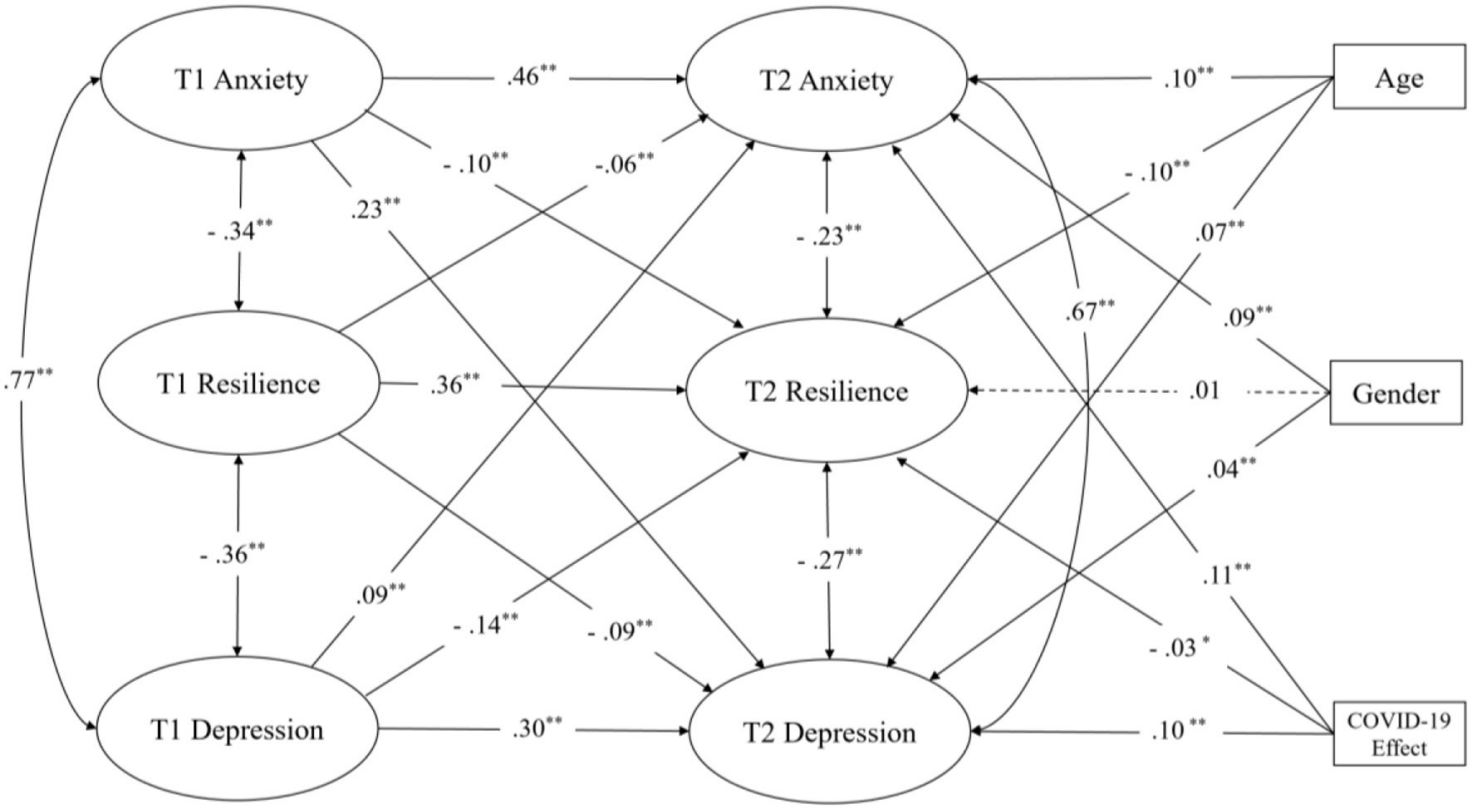
Longitudinal cross-lagged model with bidirectional effects between resilience, anxiety, and depression. Dashed lines indicate non-significant paths. **, *p* < 0.001; *, *p* < 0.05.

The longitudinal cross-lagged model presented a very good fit without controlling for covariates, namely age, gender, and COVID-19 effects (χ^2^_(2199)_ = 9874.057, *p* < 0.001, χ^2^/*df* = 4.490, CFI =0.970, NFI =0.962, TLI =0.967, RMSEA =0.021, SRMR =0.036). Additionally, the fit was also excellent when controlling for the three covariates (χ^2^_(2391)_ = 11,581.251, *p* < 0.001, χ^2^/*df* = 2391, CFI =0.965, NFI =0.956, TLI =0.961, RMSEA =0.022, SRMR =0.035). In addition, there were significant concurrent links between resilience and anxiety at both T1 (*r* = −0.34, *p* < 0.001) and T2 (*r* = −0.23, *p* < 0.001). Resilience and depression presented similar significant co-instantaneous associations at T1 (*r* = −0.36, *p* < 0.001) and T2 (*r* = −0.27, *p* < 0.001) (Figure 1).

The results showed that higher levels of resilience at T1 were significantly associated with less severe depressive (β = −0.09, *SE* = 0.01, *p* < 0.001) and anxiety symptoms (β = −0.06, *SE* = 0.01, *p* < 0.001) at T2. In contrast, they were associated with higher levels of resilience at T2 (β = 0.36, *SE* = 0.02, *p* < 0.001). Furthermore, higher depressive symptoms at T1 were significantly associated with greater depressive (β = 0.30, *SE* = 0.02, *p* < 0.001) and anxiety symptoms (β = 0.09, *SE* = 0.02, *p* < 0.001) at T2, but lower level of resilience at T2 (β = −0.14, *SE* = 0.03, *p* < 0.001). Moreover, greater anxiety symptoms at T1 were significantly associated with more severe depressive (β = 0.23, *SE* = 0.03, *p* < 0.001) and anxiety symptoms (β = 0.46, *SE* =0.03, *p* < 0.001) but lower resilience at T2 (β = −0.10, *SE* = 0.04, *p* < 0.001).

Female gender (β = 0.09, *SE* = 0.01, *p* < 0.001), older age (β =0.10, *SE* =0.002, *p* < 0.001) and greater COVID-19 impact (β = 0.11, *SE* =0.001, *p* < 0.001) were significantly associated with more severe anxiety symptoms at T2. Similarly, older age (β = 0.07, *SE* = 0.002, *p* < 0.001), female gender (β = 0.04, *SE* =0.01, *p* < 0.001), and greater COVID-19 impact (β = 0.10, *SE* = 0.001, *p* < 0.001) were significantly associated with more severe depressive symptoms at T2. In addition, being younger had a significant association with a higher level of resilience at T2 (β = −0.10, *SE* = 0.004, *p* < 0.001). More severe effect of COVID-19 was significant associated with a lower level of resilience at T2 (β = −0.03, *SE* = 0.002, *p* < 0.05). Surprisingly, gender (β = 0.01, *SE* = 0.02, *p* = 0.68) did not show a significant relationship with resilience at T2.

## Discussion

Our study may be the first empirical investigation of prospective links between resilience, depression, and anxiety based on a large longitudinal sample of Chinese adolescents before and during COVID-19. Based on a longitudinal cross-lagged SEM analysis, we found resilience at T1 (before the pandemic) negatively and significantly predicted depression and anxiety at T2. In addition, depression and anxiety at T1 negatively and significantly predicted resilience at T2 (after exposure to COVID-19). Furthermore, resilience, depression, and anxiety at T1 showed positively and significantly predictive links with resilience, depression, and anxiety at T2, respectively. Some psychosocial variables, namely age and COVID-19 impact, showed a negative predicting association with resilience at T2. The exception in this study was gender. The present study expands our understanding of the study variables relative to prior cross-sectional findings (18, 19). It provides important clarifications, illustrating the relationships between the three variables and suggesting strategies for effective resilience-related psychological interventions aimed at adolescents exposed to COVID-19.

Our study demonstrated that a higher level of resilience significantly predicted less severe depressive and anxiety symptoms during COVID-19, indicating that resilience is vital in protecting adolescents from mental health issues related to pandemic stress. This finding supports the importance of resilience as an individual's capability to “bounce back” (recover) in any crisis (2). Moreover, prior studies with adults also found that resilience was negatively associated with depression and anxiety (4, 5). One explanation is that more resilient adolescents manage psychological distress more effectively because they are more likely to have positive attitudes and optimistic beliefs when faced with life-threatening situations (38). Fergus and Zimmerman (16) proposed that resilient youth typically have positive intrapersonal traits, including good life management skills, self-efficacy, and perceived competence. Thus, resilience could buffer against the negative effect of emotional distress (e.g., depression and anxiety) despite exposure to stressors (4). Therefore, it is crucial to develop programs to improve adolescent resilience. Such programs could foster the effective prevention of mental disorders during emergencies such as the COVID-19 pandemic.

Our results indicated that the mean level of resilience showed a small yet significant reduction among Chinese adolescents after exposure to COVID-19. Consistent with previous studies, the mean level of resilience may have changed due to exposure to major public health emergencies (4). This level might be lower than that typical of Chinese adults (39). Alternatively, it may be higher than the average resilience level among Chinese patients in general (40). One possible explanation for this is that COVID-19 caused many subsequent stressful events (e.g., substantial mortality, high economic pressures, unemployment risk, and life-threatening illness). Therefore, it led to an increase in mental disorders, such as acute and post-traumatic stress disorders (ASD/PTSD) (41). Mental health problems disrupt psychological balance and reduce resilience (42). Therefore, the level of resilience might have significantly decreased among Chinese adolescents during the pandemic. Furthermore, a prior study reported that social support is essential for resilience improvement (43). Quarantine requires individuals to remain at home, leading to a decline in acquiring resources based on social support (e.g., effective information, peer contact, and community support) and decreasing resilience levels (42). Resilience improvement during COVID-19 requires further study, as it plays a significant role in psychological recovery (44). It follows that COVID-19 potentially may damage resilience and the ability to manage adverse events among adolescents during COVID-19.

The current study found a decreasing trend in the prevalence of depression and anxiety among Chinese adolescents. This finding was inconsistent with most previous research that reported mental health deterioration among those exposed to COVID-19 [e.g., (9, 45)]. However, more recent studies concluded that COVID-19 has not negatively influenced mental health. For example, a longitudinal study with a sample of 2,329 adults aged 18–65 found that the occurrence of COVID-19 did not worsen levels of anxiety and depression symptoms (46). A longitudinal observational survey of 36,520 adults in England showed a reduction in depression and anxiety symptom severity in the first 20 weeks after the implementation of a COVID-19 lockdown policy (47). A longitudinal study of 203 Chinese adolescent students revealed a significant reduction in the severity of stress, anxiety, and depression during the COVID-19 lockdown (48). A similar study of 322 adolescents in the United States reported a decreasing trend in psychological symptoms during the pandemic (13). Therefore, these results of the pandemic may not increase mental health burdens. Some factors, such as resilience, may efficiently protect youth from psychological problems. On the contrary, some potential high-risk factors could lead to mental health issues during the pandemic and should be explored and identified, such as insomnia, isolation, inadequate income, and family psychiatric history.

Some possible explanations exist for the decreased mental health burdens among adolescents exposed to COVID-19 in our study. Other factors may provide a buffer against the negative influence of COVID-19 on mental health. For example, the lockdown policy forced individuals to spend more time at home, follow a stable daily routine, and spend more time with their family, receiving more emotional support from this source. This situation may increase perceived safety and slow the risk of mental health deterioration (49). Subsequently, at-home study and online coursework may relieve academic and interpersonal relationship pressures that occur on campus. These are the primary causes of mental health symptoms among adolescent students under many conditions, including COVID-19 (50). Finally, decreasing trends for depression, anxiety, and stress could be the result of the natural restoration to the average conditions (regression to the mean) during the pandemic because of regularly changing patterns of psychological disorders during the emergency period (46).

Our results indicated that gender was not significantly associated with resilience at T2. This finding contradicts previous studies [e.g., (38, 51)]. Most prior studies reported that gender has a considerable influence on child and adolescent resilience because males and females tend to use different coping strategies (e.g., maladaptive and adaptive approaches) closely related to resilience at different developmental stages (52). Young males prefer to select adaptive coping strategies, such as direct action or positive self-instruction, when focusing on immediate issues (53). Young females may be more affected by emotional problems and tend to make use of maladaptive approaches in the face of stressors (5). Moreover, Hampel and Petermann (52) reported that females could have greater resilience in adverse situations than males because they would be more likely to seek social and emotional support. In the special period of COVID-19, male adolescents had more time at home with parents and siblings, which could have helped them to receive more social support from their families and reach the same level of resilience as females (42). This change in behavior patterns of young males may explain the lack of a significant gender difference.

## Limitations

Our study used a large sample of Chinese adolescents to track the association between resilience, depression, and anxiety longitudinally in the context of COVID-19. The results highlight the important role of resilience in reducing mental disorders, such as depression and anxiety among Chinese adolescents exposed to COVID-19. Notwithstanding these strengths, several limitations need to be noted. First, this study used a self-report questionnaire for data collection. Self-report methods may cause subjective bias in the results (35). Future studies should use a mixed-methods design, including qualitative and quantitative approaches, to comprehensively examine the effect of resilience on long-term mental health (47). Second, our study only focused on the influence of one protective factor, namely resilience. Other possible variables mitigating the impact of COVID-19 on long-term mental health problems as mediators or moderators should be investigated, including lockdown/quarantine duration, daily routine change, family support, and perceived relaxation during the pandemic (50). Finally, our study used a longitudinal cross-lagged model to demonstrate the predictive links between study variables. However, it could not establish causal relationships. Future research studies should feature experimental designs to further test the protective effect of resilience on mental issues and develop effective resilience-related interventions for adolescents (8, 54, 55).

## Conclusions

Our study found that resilience is significantly longitudinally associated with decreased mental disorders (i.e., depression and anxiety) by examining a large sample of Chinese adolescents exposed to COVID-19. These results suggest that cultivating resilience in adolescents could serve as an effective protective factor against adverse emotional effects. Moreover, our findings indicate that resilience-related interventions and programs should be developed by psychological researchers, clinical psychiatrists, social workers, mental counselors, and other psychological professionals to effectively prevent adolescents from developing mental disorders during COVID-19 and similar emergencies. Furthermore, our results suggest that resilience improvement may thwart the development of comorbidities. Low resilience is associated with other major mental disorders, such as PTSD (14). Additionally, longer-term studies should be conducted to track the development of mental health issues and uncover additional major influencing factors. These longitudinal studies could help to promote mental health recovery among adolescents during public health emergencies.

## Data availability statement

The original contributions presented in the study are included in the article/supplementary materials, further inquiries can be directed to the corresponding author/s.

## Ethics statement

The studies involving human participants were reviewed and approved by the Research Ethics Committee of the University (Registration Number: K2020025). Written informed consent to participate in this study was provided by the participants and participants' legal guardian/next of kin.

## Author contributions

PJ, LZ, and LJ designed the survey. ML and BH assisted in the data collection and cleaning. WS conducted data analysis, drafted, and submitted this manuscript. All authors contributed to the article and approved the submitted version.

## Funding

This work was supported by the Fundamental Research Funds for the Central Universities (20827044B4020), The Hong Kong Polytechnic University (19H0642), Wuhan University Specific Fund for Major School-level Internationalization Initiatives (WHU-GJZDZX-PT07), and the International Institute of Spatial Lifecourse Health (ISLE).

## Conflict of interest

The authors declare that the research was conducted in the absence of any commercial or financial relationships that could be construed as a potential conflict of interest.

## Publisher's note

All claims expressed in this article are solely those of the authors and do not necessarily represent those of their affiliated organizations, or those of the publisher, the editors and the reviewers. Any product that may be evaluated in this article, or claim that may be made by its manufacturer, is not guaranteed or endorsed by the publisher.
